# Analysis of Codon Usage Bias of 30 Chloroplast Genomes in *Ulva* (Ulvophyceae, Chlorophyta)

**DOI:** 10.3390/genes16050608

**Published:** 2025-05-21

**Authors:** Jiao Fang, Liming Qin, Hongni Liu, Zhangfeng Hu

**Affiliations:** 1 Institute of Microalgae Synthetic Biology and Green Manufacturing, School of Life Sciences, Jianghan University, Wuhan 430056, China; 02180611@cumt.edu.cn (L.Q.); liuhn0610@163.com (H.L.); 2 Hubei Engineering Research Center for Protection and Utilization of Special Biological Resources in the Hanjiang River Basin, School of Life Sciences, Jianghan University, Wuhan 430056, China; 3 Hubei Key Laboratory of Environmental and Health Effects of Persistent Toxic Substances, Jianghan University, Wuhan 430056, China

**Keywords:** *Ulva*, chloroplast genomes, codon usage pattern, natural selection, mutation pressure

## Abstract

Background: *Ulva* is a globally distributed genus with ecological and economic significance, yet the codon usage bias of the *Ulva* chloroplast genome remains poorly understood. Methods: We assessed the *Ulva* chloroplast genome codon usage patterns and their drivers by analyzing 30 genomes across 16 *Ulva* species. Results: The nucleotide composition analysis demonstrated that *Ulva* chloroplast genomes are rich in A/T, and prefer to use codons that ended with A/T. The relative synonymous codon usage analysis suggested that related species have similar codon usage patterns. A total of 25 high-frequency codons and 7–14 optimal codons were identified in these chloroplast genomes. The ENC values ranged from 31.40 to 32.76, all of which are less than 35, illustrating a strong codon bias of the *Ulva* genus. Our comparative analyses suggested that natural selection played the main role in the formation of the codon usage bias. Furthermore, the correlation analysis indicated that an influence of the base composition and gene expression levels on the codon usage bias. Conclusions: This study provides the first comprehensive analysis of the codon usage patterns in *Ulva* chloroplast genomes, improving our understanding of the genetics and evolution of these economically and ecologically important macroalgae.

## 1. Introduction

*Ulva*, a green macroalga, comprises approximately 100 taxonomically accepted species and is found in marine and freshwater environments [1,2,3]. *Ulva* species have significant ecological and economic value. For example, *Ulva* plays an important role as a primary producer in marine ecosystems, and offers a habitat for epiphytic organisms [4]. In eutrophic waters, the rapid and massive proliferation of some *Ulva* species, such as *U*. *compressa*, *U*. *prolifera*, and *U*. *meridionalis*, can eventually lead to green tides [5,6,7]. In addition, *U*. *ohnoi* and *U*. *lactuacan* can remove excess nutrients from aquaculture effluents, and therefore play an important role in algal bioremediation in aquaculture [8,9]. Furthermore, *Ulva* is often used as model organism to study the specificity of bacteria–macroalgae interactions [10,11,12]. Moreover, some species of *Ulva* show considerable potential in for use in feed and as renewable bioenergy, nutraceutical and pharmaceutical sources [13,14,15,16,17,18,19,20,21].

Codons are important bridges between DNA and proteins, and there are 64 codons in organisms [22]. The genetic code comprises 61 codons (excluding the stop codons UAA/UAG/UGA), which encode 20 amino acids. Met and Trp are encoded by single codons, while the other amino acids are encoded by 2–6 synonymous codons each; this phenomenon is referred to as codon degeneracy [23,24]. Synonymous codons refer to multiple codons that encode the same amino acid [25]. Species or genes frequently favor specific synonymous codons, creating non-uniform usage patterns termed codon usage bias (CUB) [23,26,27]. CUB regulates key cellular processes, affecting transcription, translation efficiency and accuracy, mRNA stability, and protein structure, expression, and function [26,28]. The investigation of codon usage bias is useful not only for codon optimization to increase protein expression for recombinant gene technologies, but also for understanding the evolution of organisms [29,30,31,32]. Furthermore, the study of codon usage bias is useful in revealing host–pathogen coevolution and the adaptation of pathogens to specific hosts [33,34]. Codon usage bias has been extensively studied across plants, animals, fungi, and viruses [25,35,36,37]. However, there are a few reports on the codon usage bias in *Ulva*.

Chloroplasts are the organelles that are responsible for photosynthesis. They contain their own genetic material and can transform light energy into chemical energy, which is crucial for the growth and development of plants [38,39]. Chloroplast genomes are small, uniparentally inherited, and highly conserved, which facilitates species identification, evolutionary research, and genetic engineering [40,41]. Advances in next-generation sequencing have expanded the data on *Ulva* chloroplast genomes, enabling comparative codon usage bias studies. This study aimed to investigate the *Ulva* chloroplast genome codon usage patterns and their influencing factors across 30 genomes from 16 *Ulva* species, including *U. prolifera* (4), *U. compressa* (4), *U. lacinulata* (3), *U. australis* (3), *U. torta* (2), *U. aragoënsis* (2), *U. lactuca* (2), *U. rigida* (2), *U. linza* (1), *U. californica* (1), *U*. *gigantea* (1), *U. ohnoi* (1), *U. tepida* (1), *U. meridionalis* (1), *U. intestinalis* (1), and *U. fenestrata* (1). Several indices related to codon usage bias, including the effective number of codons (ENC), the codon adaptation index (CAI), codon bias index (CBI), and relative synonymous codon usage (RSCU), were estimated, and the optimal codons for each species were determined. Furthermore, comparative analyses such as a neutrality plot, ENC plot, PR2 plot, and Correspondence analyses were conducted to explore the factors affecting the codon usage bias. This study is the first to describe the codon usage pattern of the *Ulva* genus. Our results provide insights into the genetics and molecular evolution of this ecologically and economically important genus, and contribute to improving the expression levels of exogenous genes in genetic engineering applications.

## 2. Materials and Methods

### 2.1. Sequence Collection and Processing

The 30 *Ulva* chloroplast genomes were obtained from the National Center for Biotechnology Information (NCBI) database (https://www.ncbi.nlm.nih.gov (accessed on 20 October 2023)). The analyzed chloroplast genomes included one strain each of *U. linza*, *U. californica*, *U. gigantea*, *U. ohnoi*, *U. tepida*, *U. meridionalis*, *U. intestinalis*, and *U. fenestrata*; two strains each of *U. torta*, *U. aragoënsis, U. lactuca*, and *U. rigida*; three strains each of *U. lacinulata* and *U. australis*; and four strains each of *U. prolifera* and *U. compressa*. Detailed information about the 30 *Ulva* chloroplast genomes is provided in Table S1.

MAGE version 6 was employed to process the original CDSs [42]. The CDSs were filtered according to the following criteria: the sequence length must be at least 300 bp and the CDSs must start with the start codon (ATG) and end with a stop codon (TAA, TAG, or TGA). Additionally, CDSs must not contain any internal stop codons or ambiguous nucleotides, and the sequence lengths must be divisible by three [25,43,44]. The number of filtered sequences ranged from 37 to 46 for the 30 *Ulva* chloroplast genomes (Table S1); these sequences were used for further analysis.

### 2.2. Codon Usage Indices

CodonW version 1.4.2 was used to calculate A3, T3, C3, and G3, which stand for the A, T, C, and G contents at the 3rd position of codons, respectively. Additionally, GC3s, which indicates G and C content at the 3rd position of synonymous codons, was also calculated. The online CUSP tool (https://bioinformatics.nl/cgi-bin/emboss/cusp, accessed on 10 November 2023) was used to determine the overall GC content, as well as GC1, GC2, and GC3. GC12 denotes the mean value of GC1 and GC2. CodonW version 1.4.2 was utilized to calculate the codon adaptation index (CAI), codon bias index (CBI), and effective number of codons (ENC). The relative synonymous codon usage (RSCU) values were acquired using the online JSHYCloud platform (http://cloud.genepioneer.com:9929, accessed on 10 November 2023). The codon usage bias analysis included 59 synonymous codons for 18 amino acids, with exclusions for Met (AUG), Trp (UGG), and the stop codons (UAG, UAA, and UGA) [45].

### 2.3. Identification of Optimal Codon

The genes were initially sorted by their ENC value; the highest and lowest 10% were designated as the high/low-expression groups. We then computed and contrasted their RSCU values using ΔRSCU (ΔRSCU = RSCUhigh − RSCUlow). Finally, codons that met the criteria of RSCU > 1 and ΔRSCU > 0.08 were identified as optimal codons [37].

### 2.4. ENC Plot Analyses

ENC is a straightforward measure of overall codon bias, with values ranging from 20 to 61 [46]. The ENC value decreases with an increasingly uneven use of codons. A value of 20 indicates that for each amino acid, only one codon is employed, while a value of 61 suggests that all codons are utilized with equal frequency [47]. A strong codon usage bias corresponds to ENC values below 35, with values above this threshold indicating a weaker bias.

The ENC-GC3s plot (GC3 on x-axis, ENC on y-axis) was used to identify mutation-driven versus selection-driven codon usage. The standard curve was determined as follows:

(1) $${\mathrm{ENC}}_{\exp}=2+\mathrm{GC}3s+\frac{29}{GC3s+{\left(1-GC3s\right)}^{2}}$$

ENC_exp_ denotes the expected ENC. Points on the curve suggest mutation-driven codon bias, while deviations imply selection influence [48].

The ENC_ratio_ quantifies the deviations between expected and observed ENC values:

(2) $${\mathrm{ENC}}_{\mathrm{ratio}}=\frac{{\mathrm{ENC}}_{\exp}-{\mathrm{ENC}}_{\mathrm{obs}}}{{\mathrm{ENC}}_{\exp}}$$

The ENC_obs_ values were computed using CodonW version 1.4.2.

### 2.5. PR2 Plot Analyses

A Parity Rule 2 (PR2) plot, which was constructed with A3/(A3 + T3) on the y-axis and G3/(G3 + C3) on the x-axis, was used to assess the degree and direction of gene bias. The reference lines mark X = 0.5 and Y = 0.5. Without bias, points will cluster at the center (A3 = T3, G3 = C3) [49]. In theory, if CUB is driven exclusively by mutational pressure, the frequency of A/T and G/C should be equal. However, if they differ, it suggests that the codon bias was shaped by natural selection [22]. Significant deviations from the center point in the PR2 plot are primarily attributed to selective bias rather than mutational bias between the two DNA strands [50].

### 2.6. Neutrality Plot Analyses

A neutral plot was generated to estimate the relative contributions of mutation and selection in genes. A scatter plot was generated, with GC12 on the x-axis and GC3 on the y-axis. GC12 denotes the mean of GC1 and GC2. A slope close to 1 indicates mutation-driven codon bias, while a slope close to 0 suggests selection-dominated bias [51,52].

### 2.7. Correspondence Analyses

Correspondence analysis (COA), a standard multivariate method, can identify the sources of major variations in codon usage patterns [53]. Correspondence analysis generates a series of orthogonal axes to identify trends that account for the variation in the data, with each successive axis explaining a diminishing amount of variation. The codon usage patterns were analyzed using 59 synonymous codons (excluding Met and Trp), resulting in the generation of up to 58 axes. Axis 1 explains the largest codon usage variation, with diminishing contributions from the remaining 58 axes. To further investigate the factors influencing codon usage bias, a correlation analysis was conducted to examine the correlation between Axis 1 and GC1, GC2, GC3, GC3s, CAI, CBI, ENC, and L_aa.

## 3. Results

### 3.1. Nucleotide Composition

**Figure 1:**
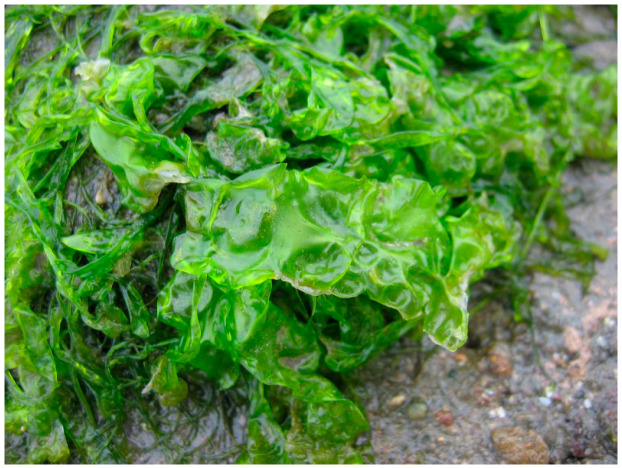
Morphology of *U*. *lactuca* (Image credit: Kristian Peters, Wikimedia Commons, licensed under CC BY-SA 3.0; links to licenses for the photograph: https://commons.wikimedia.org/wiki/File:Ulva_lactuca.jpeg, accessed on 10 May 2025).

Figure 1 shows the representative morphology of *Ulva* species, displaying the characteristic thin, sheet-like thallus structure of this genus. The accession numbers for the 30 *Ulva* chloroplast genomes are listed in Table S1. The number of filtered sequences varied from 37 to 46. A3%, T3%, C3%, G3%, GC%, GC1%, GC2%, and GC3% were calculated. As was shown in Table 1, the GC% values of all 30 chloroplast genomes were below 50%, indicating a bias towards A/T bases in *Ulva*. *U. compressa* (MK069584) exhibited the highest GC content at 28.95%, while *U. australis* (MT179348) displayed the lowest GC content at 23.70%. A3% ranged from 40.91% to 43.32% (average: 42.70%), T3% ranged from 47.85% to 50.08% (average: 49.22%), and C3% ranged from 4.38% to 6.68% (average: 5.10%), while G3% values ranged from 2.50% to 3.75%, with an average of 2.97%, which was the lowest average. The frequency of nucleotides at the third codon position followed the order T3% > A3% > C3% > G3%, indicating that a preference for A/T-ending codons in *Ulva*. The GC content in the three codon positions was unbalanced, with a distribution trend of GC1 > GC2 > GC3. It was observed that the GC3 values for all the investigated chloroplast genomes were less than 50%, further suggesting a preference for A/T-ending codons in *Ulva*.

### 3.2. The Relative Synonymous Codon Usage and Optimal Codons

**Figure 2:**
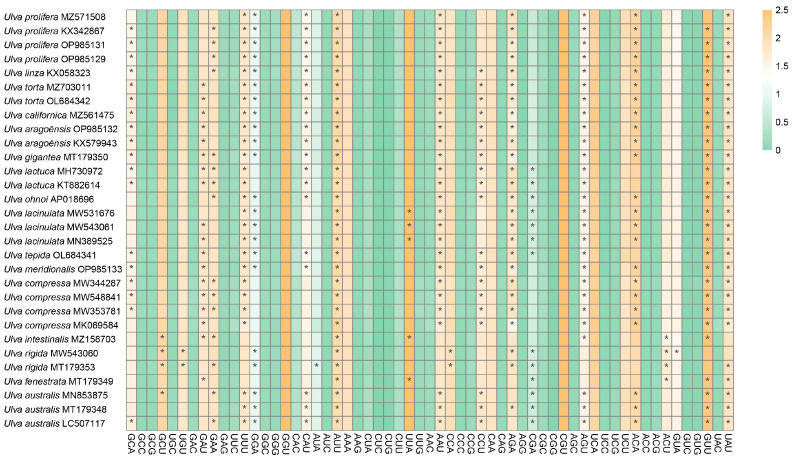
A heat map illustrating the RSCU values of the 30 *Ulva* chloroplast genomes. The color gradient transitions from green to yellow signify increasing RSCU values. Optimal codons, defined as having a ΔRSCU > 0.08 and RSCU > 1, are indicated with an asterisk (*).

A total of 59 codons (excluding stop codons and AUG/UGG) were analyzed in this study. The 30 chloroplast genomes of *Ulva* showed highly conserved codon usage (Figure 2). There are 25 high-frequency codons (RSCU > 1) that are shared among these species. Among these 25 codons, 15 end with U, 10 end with A, and no synonymous codons end with C or G, indicating a bias toward A/U-ending codons.

The RSCU analysis showed that *Ulva gigantea* possesses the most optimal codons, 14 in total, while *Ulva fenestrata* has the lowest, with only 7. Thirteen optimal codons were identified in *U. linza*, *U. torta* MZ703011, *U. torta* OL684342, *U. californica*, *U. aragoënsis* OP985132, *U. aragoënsis* KX579943, *U. lactuca* MH730972, *U. lactuca* KT882614, *U. ohnoi*, *U. tepida*, *U. meridionalis*, *U. australis* MN853875, and *U. australis* LC507117. Twelve optimal codons were identified in *U. prolifera* KX342867, *U. prolifera* OP985131, *U. prolifera* OP985129, *U. lacinulata* MW543061, *U. lacinulata* MN389525, *U. compressa* MW344287, *U. compressa* MW548841, *U. compressa* MW353781, and *U. australis* MT179348. A total of 11 optimal codons were identified in *U. lacinulata* MW531676 and *U. rigida* MT179353, followed by 10 optimal codons in *U. compressa* MK069584. Both *U. prolifera* MZ571508 and *U. rigida* MW543060 have 9 optimal codons, and *U. intestinalis* have 8 optimal codons. Notably, all of these optimal codons end with A or U, and AUU is the most frequently used optimal codon in all 30 *Ulva* chloroplast genomes.

### 3.3. ENC Plot Analyses

ENC measures the strength of codon usage bias, and serves as the most informative indicator for assessing codon usage bias. The ENC values varied from 20 to 61. An ENC value equal to 20 indicates that there is a unique codon for each amino acid, and 61 indicates that all synonymous codons are used equally. A lower ENC value indicates a stronger codon bias. Generally, ENC < 35 indicates a strong bias. In the 30 *Ulva* chloroplast genomes, most genes exhibited ENC values below 35, and the average ENC values ranged from 31.40 to 32.76, indicating a strong codon usage bias among *Ulva* strains (Table 2).

**Figure 3:**
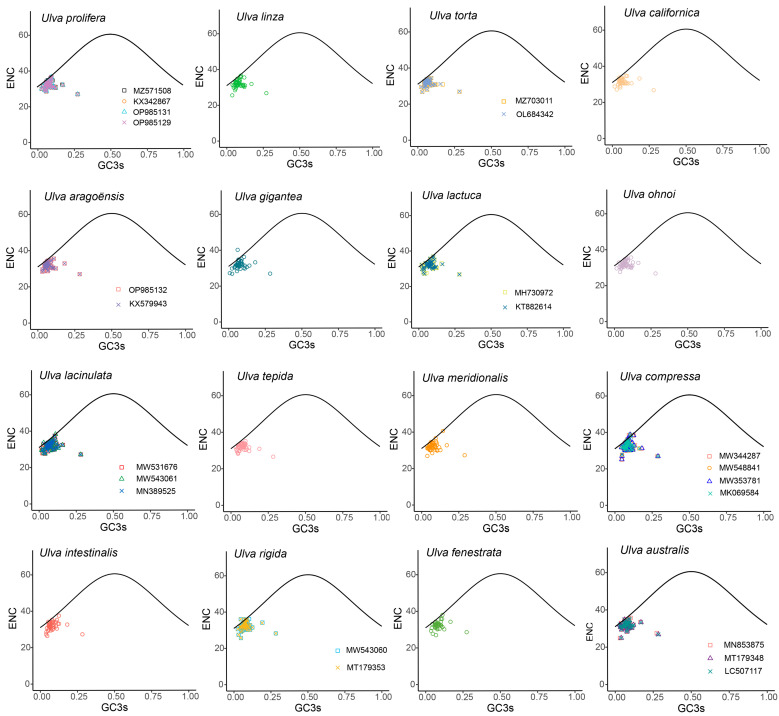
ENC plot of 30 *Ulva* chloroplast genomes. The black curve represents the predicted gene distribution under mutation pressure.

An ENC-GC3s plot is commonly adopted to evaluate whether the codon usage bias is affected by mutation pressure or natural selection. To investigate the factors affecting CUB in *Ulva*, an ENC-GC3s plot was drawn (Figure 3). The ENC plot revealed that most genes fall below the standard curve, with few genes on or near the standard curve (Figure 3). The results showed that *Ulva* chloroplast CUB was shaped by both mutation pressure and natural selection, with natural selection having a stronger influence.

**Figure 4:**
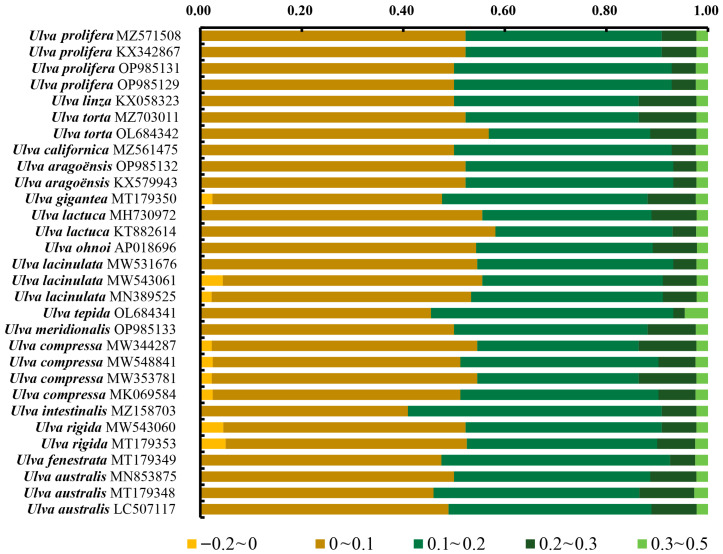
Frequency distribution of ENCratio. Most genes showed a ratio of 0.1–0.2.

To further investigate the discrepancies between the observed and expected ENC values, the ENC_ratio_ ((ENC_exp_ − ENC_obs_)/ENC_exp_) was derived (Figure 4). Most genes showed ENC ratios of 0–0.2, reflecting consistently lower observed than expected ENC values. The results of this analysis were consistent with those presented in Figure 3.

### 3.4. PR2 Plot Analyses

**Figure 5:**
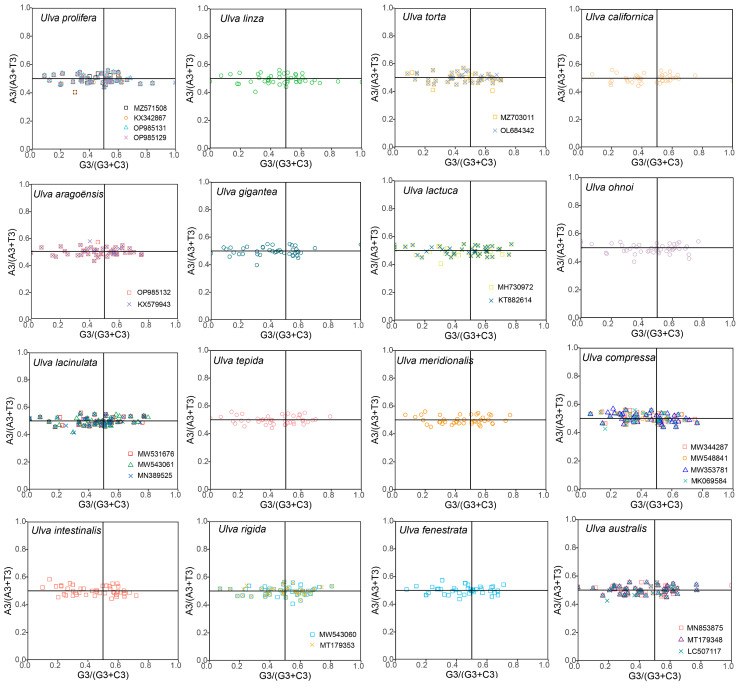
PR2 plot of 30 *Ulva* chloroplast genomes. The horizontal axis denotes the value of G3/(G3 + C3) and the vertical axis denotes the value of A3/(A3 + T3).

PR2 analysis was conducted to estimate the effect of natural selection and mutation pressure on the genes from the 30 *Ulva* chloroplast genomes (Figure 5). Theoretically, if codon usage bias was solely affected by mutation pressure, the genes would be evenly distributed around the center point. Conversely, if genes are unevenly distributed in the four quadrants, the codon usage bias was determined by natural selection. Figure 5 shows that most genes clustered along the horizontal axis with an uneven quadrant distribution, reflecting strong selection shaping *Ulva* chloroplast CUB.

### 3.5. Neutrality Plot Analyses

**Figure 6:**
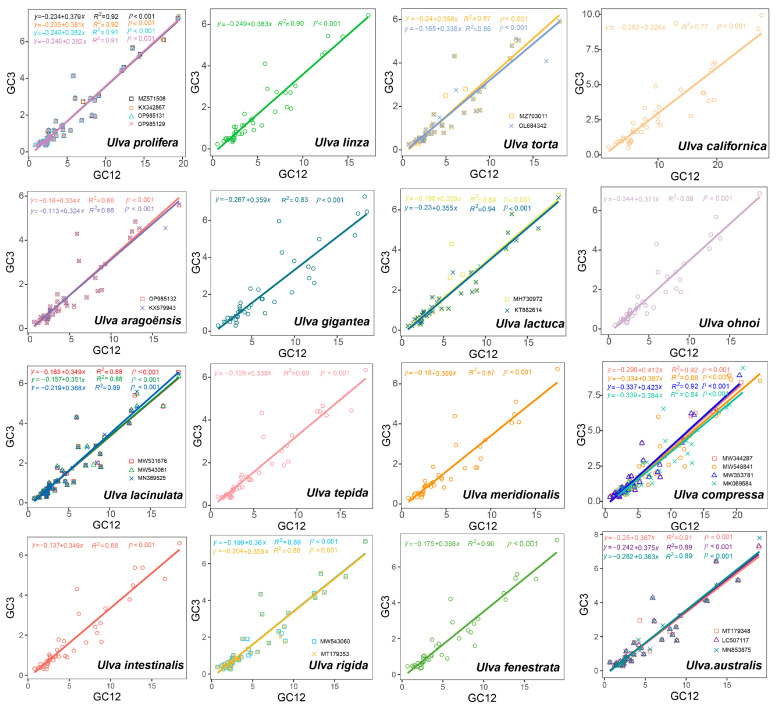
Neutrality plot of 30 *Ulva* chloroplast genomes. Diagonal solid line: regression line (equation shown).

The neutrality plot analyses quantified the mutation pressure and natural selection effects (Figure 6). Figure 6 shows that the regression coefficients were between 0.324 and 0.423, indicating that natural selection predominantly shaped the codon usage in the 30 *Ulva* chloroplast genomes. The average regression coefficient for the 30 strains was 0.37, suggesting that mutation pressure accounts for 37% of the influence on CUB in the 30 *Ulva* chloroplast genomes, while natural selection accounts for the remaining 63%. Consequently, the natural selection effect dominates over that of mutation pressure in *Ulva*.

### 3.6. Correspondence Analyses

**Figure 7:**
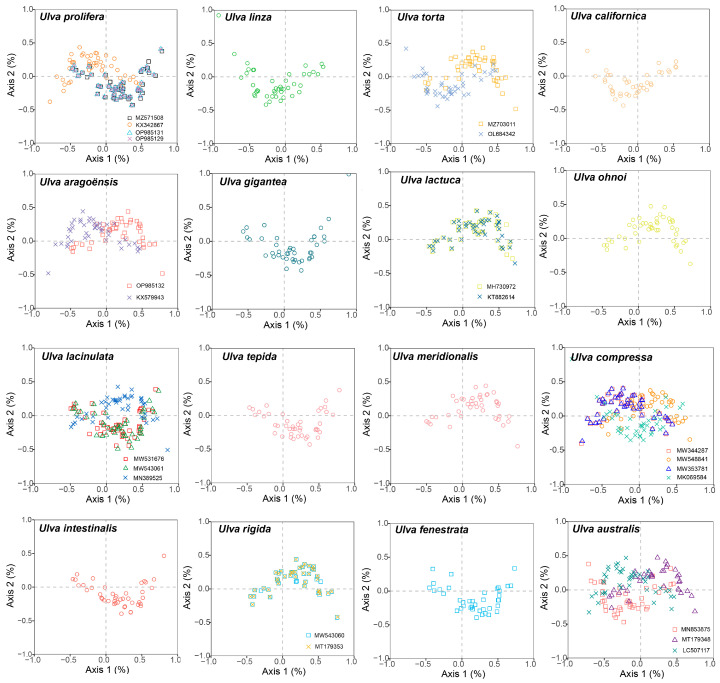
Correspondence analyses of the 30 *Ulva* chloroplast genomes.

COA was conducted using the RSCU values from the 30 *Ulva* chloroplast genomes. A total of 59 codons (excluding TGG, which encodes Trp; ATG, which encodes Met; and the three stop codons TGA, TAG, and TAA) were utilized to generate the orthogonal axes, which reflect the diversity of codon usage. Axis 1 captures the primary codon usage variation, with diminishing contributions from the subsequent axes. The synonymous codons were found to be distributed across four regions, with the majority predominantly aligned along Axis 1 (Figure 7). Axis 1 explained 4.24% to 20.43% of the total variation in the genes of the 30 *Ulva* chloroplast genomes, whereas Axis 2 explained 6.60% to 9.40% of the variation. This indicates that Axis 1 shows the dominant impacts, followed by Axis 2.

We correlated Axis 1 with key codon indices, including GC1, GC2, GC3, CAI, and ENC. Axis 1 demonstrated a significant correlation with GC3 and GCall in all 30 *Ulva* chloroplast genomes (*p* < 0.001), indicating an influence of the base composition on the codon usage bias (Figure S1). Moreover, Axis 1 showed a significant correlation with CAI (*p* < 0.001) in all the *Ulva* chloroplast genomes, suggesting that gene expression levels might shape the codon usage bias (CUB) of *Ulva*. Additionally, GC3 exhibited a significant positive correlation with both GC1 and GC2 (*p* < 0.001), and a significant correlation was also observed between GC1 and GC2 (*p* < 0.001).

## 4. Discussion

CUB reflects non-random synonymous codon usage, which is prevalent in bacteria [54], viruses [55,56], fungi [57], and plants [58,59]. CUB plays a crucial role in determining gene expression levels and cellular functions, including RNA processing, protein translation, and protein folding [60,61,62]. Investigating codon usage bias patterns contributes to the understanding of the genetic architecture and evolution of organisms [63]. For example, the codon usage bias in chloroplast genomes of Zingiberaceae species from different habitats reflects habitat-specific adaptive evolution, particularly the light adaptation genes that show differential expression between species from different habitats [51]. In addition, the study of CUB has important implications in genetic engineering, particularly in transgenic plants. A study investigating the effects of codon optimization on the expression of bacterial *bar* genes in transgenic tobacco revealed that a certain proportion of optimal codons can enhance transgene expression levels [64]. Host codon optimization enhances heterologous gene expression and product yield [25,32]. *Ulva* species have important economic and ecological value; however, the characteristics of the codon usage in their chloroplast protein-coding genes have not been fully elucidated. Therefore, we conducted an analysis of the CUB in 30 *Ulva* chloroplast genomes, and investigated the factors influencing CUB. Our result elucidated the codon usage and evolutionary traits of this green macroalgal genus.

In this study, A and T nucleotides exhibited significantly greater abundance compared to G and C at the 3rd codon position (Table 1). This finding suggests a bias for A/T bases. Additionally, the GC3 content is less than 50%, further indicating that *Ulva* species prefer A/T-ending codons. Furthermore, 25 codons with an RSCU > 1 and all optimal codons terminated with A/T further highlight a bias for A/T bases at the 3rd codon position. Our findings are consistent with those of previous studies on *Camellia*, cherries, and Rosales, which also prefer to use codons ending in A/T [65,66,67]. The total GC contents of the 30 *Ulva* chloroplast genome range from 23.7 to 28.95 (Table 1), indicating a strong preference for A/T bases in *Ulva* chloroplast genomes. A strong A/T bias was also found in the chloroplast genomes of other Ulvophyceae groups such as *Blidingia marginata* [68], *Trentepohlia* [69], *Cephaleuros* [69], and Halimedineae [70], showing that closely related Ulvophyceae taxa have similar codon usage biases. This A/T bias in chloroplast genomes is absent from *Ulva* nuclear genomes [71,72]. Considering that AT synthesis requires two high energy phosphate bonds, while GC synthesis requires three, the observed A/T bias in *Ulva* chloroplast genomes may reduce energy consumption, which likely conferred *Ulva* species with an adaptive advantage in aquatic environments. Optimal codons enhance translational efficiency and accuracy, increasing expression levels [73]. Therefore, optimal codon analysis is essential for enhancing heterologous gene expression. The comparative analysis showed that a total of 25 codons with an RSCU > 1 are common in the 30 *Ulva* chloroplast genomes. Furthermore, we identified 7–14 optimal codons for each species, and AUU is the most frequently used optimal codon in all 30 *Ulva* chloroplast genomes. In Halimedineae, a close relative of *Ulva*, the types and frequency of optimal codons differed from those of *Ulva*. The most commonly used optimal codons in Halimedineae are UUU, CAU, CUU, CCA, AGA, CGA, and AGU [70], while in *Ulva*, they are UUU, GGA, GAU, AUU, AAU, AGA, AGU, ACA, GUU, and UAU. This difference may be due to their ecological adaptations. In summary, our results have significant implications for the optimization of gene modifications and provide a reference for designing stable and efficient gene expression constructs.

ENC is a crucial parameter for assessing codon usage bias in genes. The ENC value ranges from 20, which indicates that only one codon is effectively utilized for each amino acid, to 61, which suggests that codons are employed equally [67]. A lower ENC value signifies a stronger codon usage bias, whereas a higher value implies a weaker bias. An ENC value < 35 indicates a strong codon bias [25]. In this study, most genes exhibited ENC values below 35, and the average ENC value, which ranged from 31.40 to 32.76, was also less than 35 in all 30 *Ulva* chloroplast genomes, suggesting a strong codon bias for chloroplast genes of *Ulva* (Table 2). In the analysis of the codon usage bias in Gracilariaceae chloroplast genomes, the average ENC values ranged from 35.098 to 42.327, which were all above 35 [74]. Compared with the average ENC value of 31.40 to 32.76 in *Ulva* in this study, there is a relatively weak bias, indicating the significant variation in codon usage patterns among different algal taxa.

Codon usage bias can be influenced by various factors, including mutational pressures, natural selection, the base composition of genes, the gene lengths, the gene expression levels, amino acid hydrophobicity, the tRNA abundance, and the aromaticity [75,76]. Among these factors, natural selection and mutation pressure are the most significant. Previous studies demonstrated that natural selection was the primary factor shaping the codon usage bias across diverse plant lineages, including Rutaceae [25], *Aconitum* [44], *Oryza* [52], Euphorbiaceae [77], and *Pisum* [78]. This is consistent with the results from this study. Natural selection was found to have played an important role in shaping the codon usage patterns of *Ulva*, as evidenced by the ENC plot analyses, PR2 plot analyses, and neutrality plot analyses. In the ENC plot analyses, almost all of the genes were located below the expectation curve (Figure 3), indicating that natural selection played an important role in the formation of the codon usage bias. The unequal usage frequencies of the four bases in the PR2 plot also indicated that natural selection contributed to the codon usage patterns (Figure 5). In addition, the neutrality plot analyses revealed regression coefficients ranging from 0.324 to 0.423 (approaching 0), demonstrating that mutation pressure played a small role in the formation of the codon bias, and natural selection predominantly shaped the codon usage bias in the 30 *Ulva* chloroplast genomes (Figure 6). *Ulva*, as an aquatic green macroalga, lives in complex and variable aquatic environments. Natural selection may promote its adaptation to environmental changes by regulating the codon bias of genes related to photosynthesis and nutrient absorption [79,80]. Furthermore, we detected correlations between Axis 1 and nucleotide composition (GC1, GC2, GC3, and GCall), CAI, CBI, ENC, and L_aa (the length of the amino acid sequence). These findings indicate the influence of the base composition and gene expression levels on CUB. In conclusion, this study analyzed the codon usage characteristics of 30 *Ulva* chloroplast genomes and enhanced our understanding of the codon usage characteristics and genetic evolution of this green macroalgal genus. Future research is needed to validate whether the identified optimal codons enhance translation efficiency in *Ulva* species.

## 5. Conclusions

In this study, the codon usage patterns and influencing factors in 30 *Ulva* chloroplast genomes were analyzed. The nucleotide composition analysis indicated that *Ulva* species tend to use A/U bases and codons that ended with A/U. Furthermore, we identified the optimal codons for each species, all of which also end in A/U. Functional experiments are needed to validate whether the identified optimal codons enhance translation efficiency in *Ulva* species. The relative synonymous codon usage (RSCU) analyses demonstrated that the codon usage patterns of closely related species are conserved. Additionally, the average ENC values of the 30 *Ulva* chloroplast genomes ranged from 31.40 to 32.76, indicating a stronger bias in *Ulva* chloroplast genomes. Future studies should investigate the codon usage characteristics of the *Ulva* mitochondrial genome and nuclear genome to determine the differences in codon usage patterns between organelle genomes and the nuclear genome. The factors influencing the formation of the codon usage patterns were analyzed, and our study results suggested that natural selection was the primary factor driving the codon usage bias. Furthermore, the correlation analysis indicated an influence of the base composition and gene expression levels on the codon usage bias. Our results provide a foundation for developing specific codon optimization tools for *Ulva* chloroplast engineering, which could significantly improve transgene expression in ecologically and economically important *Ulva* species.

## Figures and Tables

**Table 1:** Nucleotide composition of 30 *Ulva* chloroplast genomes.

Species	Accession Number	A3%	T3%	C3%	G3%	GC%	GC1%	GC2%	GC3%
*Ulva prolifera*	MZ571508	42.41	49.25	5.10	3.23	24.60	35.46	30.00	8.33
*Ulva prolifera*	KX342867	42.41	49.37	4.98	3.24	24.60	35.47	29.95	8.37
*Ulva prolifera*	OP985131	42.21	49.48	5.17	3.13	24.75	35.87	30.07	8.30
*Ulva prolifera*	OP985129	42.21	49.48	5.55	2.75	24.75	35.87	30.07	8.30
*Ulva linza*	KX058323	42.83	48.50	5.39	3.28	26.13	38.09	31.63	8.67
*Ulva torta*	MZ703011	42.46	50.08	4.88	2.58	24.79	36.45	30.47	7.46
*Ulva torta*	OL684342	43.29	49.60	4.44	2.67	25.00	35.92	30.39	8.68
*Ulva californica*	MZ561475	43.32	49.31	4.87	2.50	26.70	37.35	33.39	9.35
*Ulva aragoënsis*	OP985132	42.88	49.96	4.53	2.64	24.24	35.78	29.77	7.16
*Ulva aragoënsis*	KX579943	43.26	49.69	4.38	2.67	23.84	34.58	27.78	9.16
*Ulva gigantea*	MT179350	42.79	49.06	5.40	2.75	26.59	39.28	30.77	9.73
*Ulva lactuca*	MH730972	42.72	49.47	4.85	2.96	25.34	36.19	30.65	9.18
*Ulva lactuca*	KT882614	42.93	49.34	4.81	2.91	23.91	34.75	29.25	7.73
*Ulva ohnoi*	AP018696	42.66	49.54	4.82	2.98	24.37	35.55	29.74	7.80
*Ulva lacinulata*	MW531676	43.03	49.14	4.94	2.90	24.81	36.17	30.42	7.84
*Ulva lacinulata*	MW543061	42.78	49.48	4.85	2.89	24.18	35.23	29.58	7.74
*Ulva lacinulata*	MN389525	42.75	49.46	4.88	2.91	24.20	35.25	29.56	7.79
*Ulva tepida*	OL684341	43.09	49.46	4.71	2.74	23.79	34.67	29.26	7.45
*Ulva meridionalis*	OP985133	42.60	49.63	4.94	2.84	24.28	35.40	29.67	7.77
*Ulva compressa*	MW344287	42.12	48.06	6.06	3.75	25.22	36.08	29.77	9.81
*Ulva compressa*	MW548841	43.08	47.85	5.84	3.23	25.91	37.56	31.11	9.07
*Ulva compressa*	MW353781	42.33	48.65	5.50	3.52	24.72	35.56	29.58	9.02
*Ulva compressa*	MK069584	42.56	47.94	6.37	3.13	28.95	42.77	34.58	9.50
*Ulva intestinalis*	MZ158703	42.91	49.32	4.89	2.87	24.01	34.99	29.29	7.76
*Ulva rigida*	MW543060	42.86	49.37	4.68	3.09	23.98	34.88	29.31	7.77
*Ulva rigida*	MT179353	42.77	49.52	4.67	3.04	23.98	34.85	29.37	7.71
*Ulva fenestrata*	MT179349	42.70	49.17	5.06	3.07	24.04	34.66	29.32	8.14
*Ulva australis*	MN853875	42.90	49.38	4.77	2.94	24.05	35.11	29.34	7.71
*Ulva australis*	MT179348	40.91	49.47	6.68	2.93	23.70	34.29	28.92	7.88
*Ulva australis*	LC507117	43.28	48.64	5.06	3.02	25.58	35.51	33.14	8.08

**Table 2:** Summary table of ENC values of 30 *Ulva* chloroplast genomes.

Species	Accession Number	Average ENC Value	Gene Number	
			ENC < 35	35 ≤ ENC ≤ 61
*Ulva prolifera*	MZ571508	32.22	41	3
*Ulva prolifera*	KX342867	32.20	41	3
*Ulva prolifera*	OP985131	32.11	38	4
*Ulva prolifera*	OP985129	32.11	38	4
*Ulva linza*	KX058323	32.01	40	4
*Ulva torta*	MZ703011	31.43	44	0
*Ulva torta*	OL684342	31.56	44	0
*Ulva californica*	MZ561475	31.40	42	0
*Ulva aragoënsis*	OP985132	31.58	43	1
*Ulva aragoënsis*	KX579943	31.62	43	1
*Ulva gigantea*	MT179350	31.84	40	2
*Ulva lactuca*	MH730972	31.91	41	4
*Ulva lactuca*	KT882614	32.02	39	4
*Ulva ohnoi*	AP018696	31.83	43	3
*Ulva lacinulata*	MW531676	31.94	41	3
*Ulva lacinulata*	MW543061	31.95	42	3
*Ulva lacinulata*	MN389525	31.94	42	3
*Ulva tepida*	OL684341	31.84	43	1
*Ulva meridionalis*	OP985133	32.10	40	2
*Ulva compressa*	MW344287	32.74	36	8
*Ulva compressa*	MW548841	32.57	34	7
*Ulva compressa*	MW353781	32.76	37	7
*Ulva compressa*	MK069584	32.71	33	8
*Ulva intestinalis*	MZ158703	31.78	41	3
*Ulva rigida*	MW543060	32.22	39	5
*Ulva rigida*	MT179353	32.11	36	4
*Ulva fenestrata*	MT179349	32.19	37	3
*Ulva australis*	MN853875	31.81	42	2
*Ulva australis*	MT179348	31.43	37	0
*Ulva australis*	LC507117	31.80	44	1

## Data Availability

The original contributions presented in the study are included in the article/Supplementary Materials; further inquiries can be directed to the corresponding author.

## Supplementary Materials

The following supporting information can be downloaded at: https://www.mdpi.com/article/10.3390/genes16050608/s1, Table S1: Accession numbers of 30 *Ulva* chloroplast genomes; Figure S1: Correlation matrix of codon usage indices across 30 *Ulva* strains. Gradient colors (yellow→green) denote increasing strengths of correlations (* *p* < 0.05, ** *p* < 0.01, *** *p* < 0.001).
