# Supplementary material for: Analysis of Codon Usage Bias of 30 Chloroplast Genomes in Ulva (Ulvophyceae, Chlorophyta)
Source: Genes (Basel). 2025 May 21;16(5):608. doi: 10.3390/genes16050608 (PMC12111487; doi:10.3390/genes16050608)
Supplement: Supplementary file 1 [file genes-16-00608-s001.zip › Supplementary Figure S1.pdf]

Ulva prolifera MZ571508

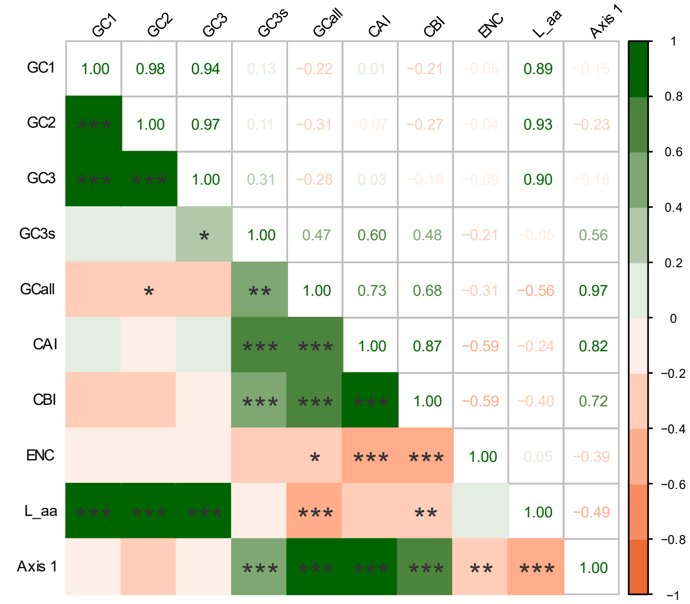

Ulva prolifera KX342867

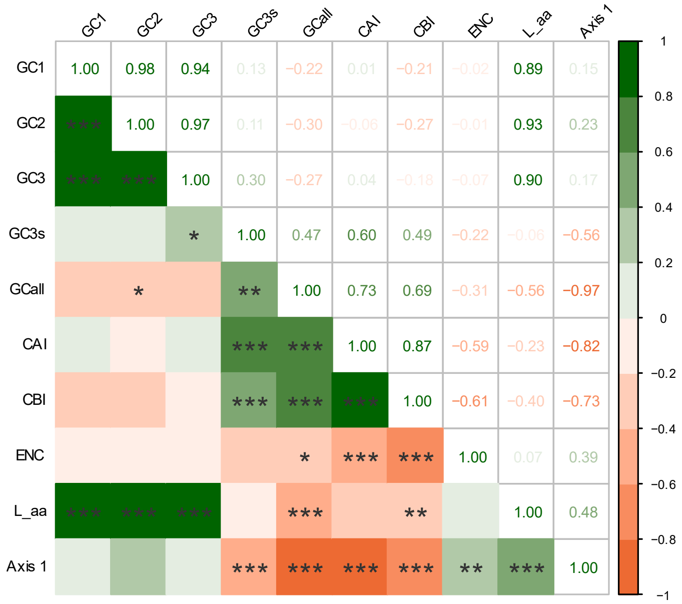

Ulva prolifera OP985131

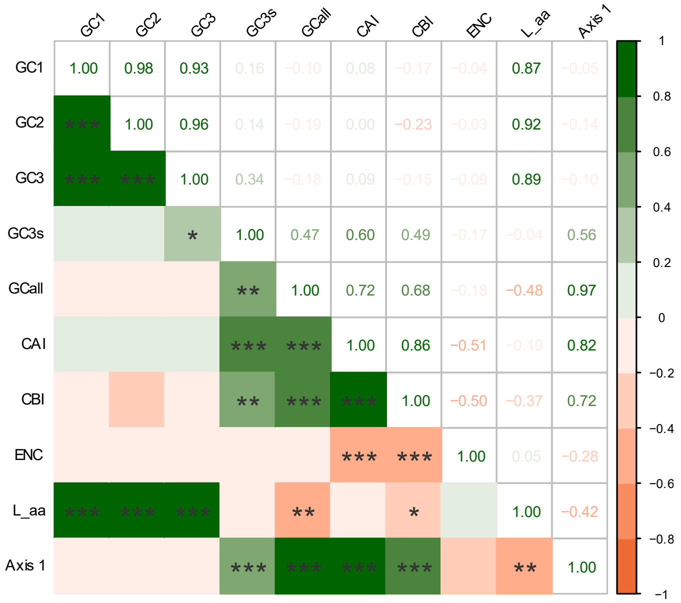

Ulva prolifera OP985129

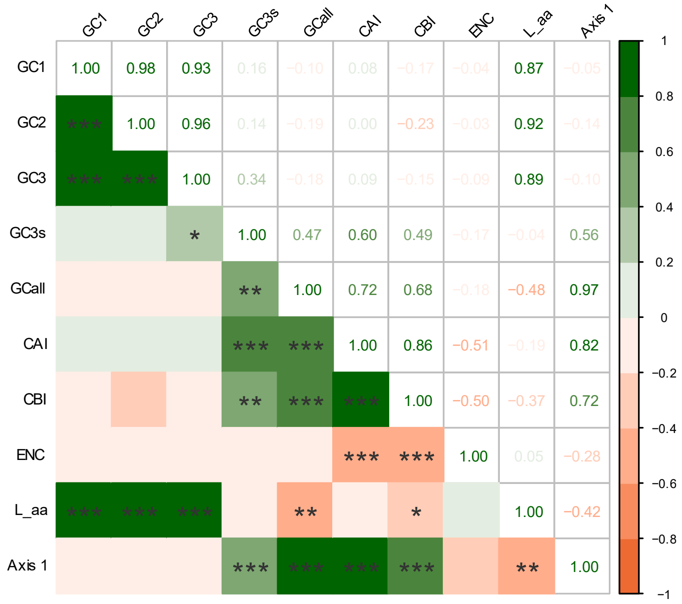

Ulva linza KX058323

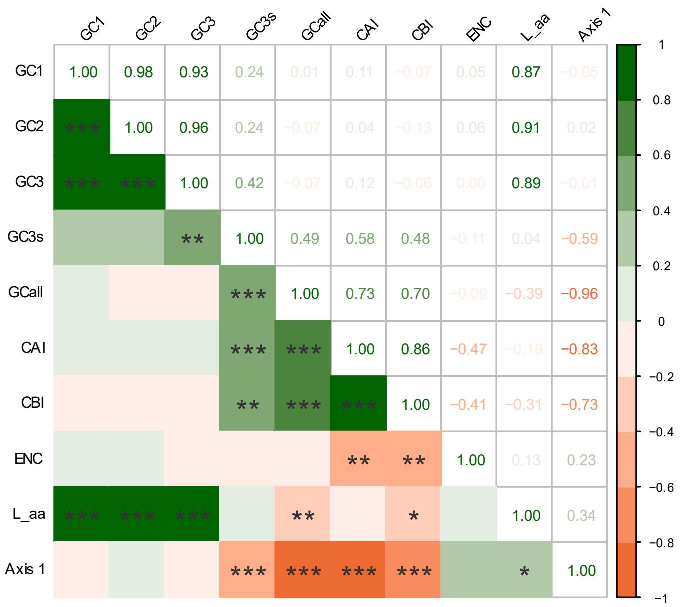

Ulva torta MZ703011

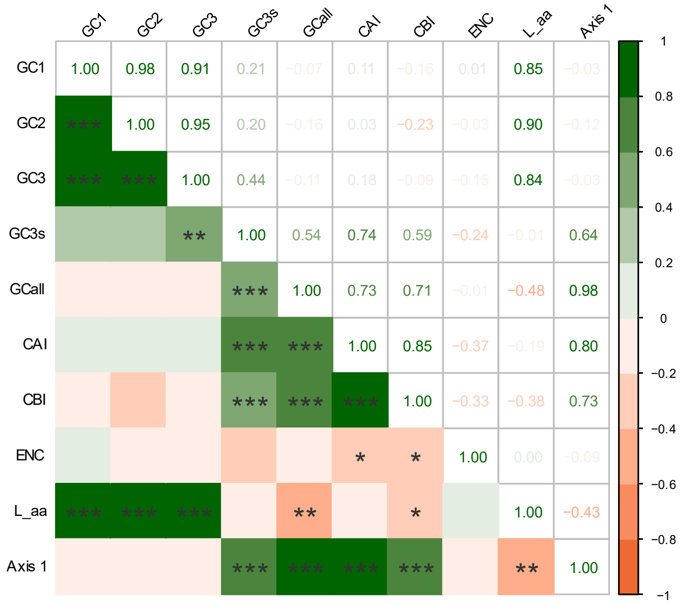

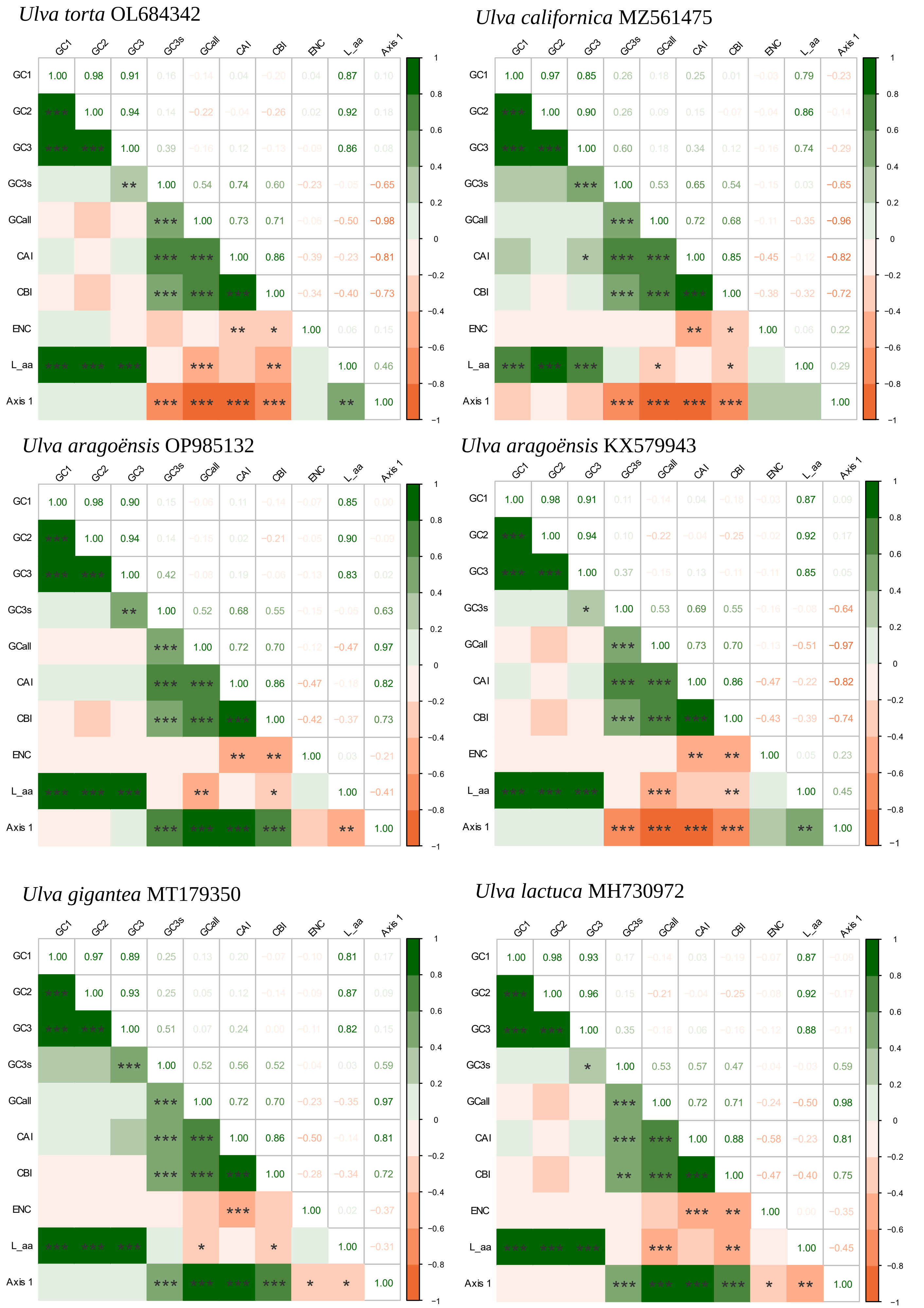

Ulva torta OL684342
Ulva californica MZ561475
Ulva aragoënsis OP985132
Ulva aragoënsis KX579943
Ulva gigantea MT179350
Ulva lactuca MH730972
GC1
GC2
GC3
GC3s
GCall
CAI
CBI
ENC
L_aa
Axis 1

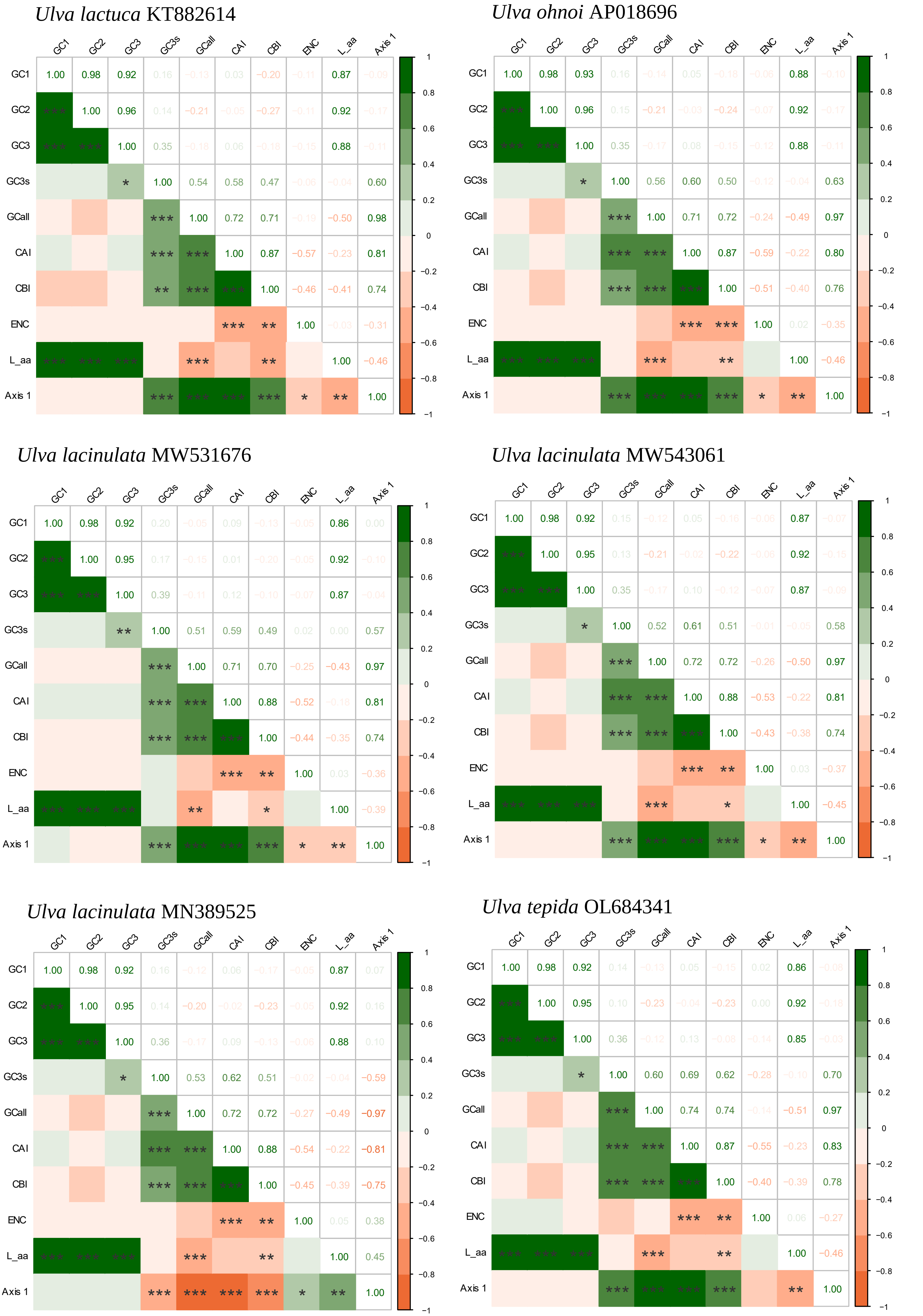

Ulva lactuca KT882614
Ulva ohnoi AP018696
Ulva lacinulata MW531676
Ulva lacinulata MW543061
Ulva lacinulata MN389525
Ulva tepida OL684341
GC1
GC2
GC3
GC3s
GCall
CAI
CBI
ENC
L_aa
Axis 1

Ulva meridionalis OP985133

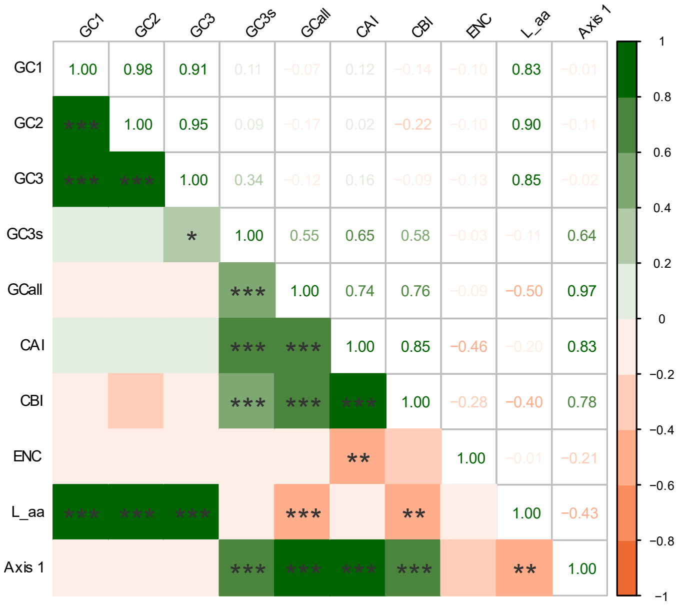

Ulva compressa MW344287

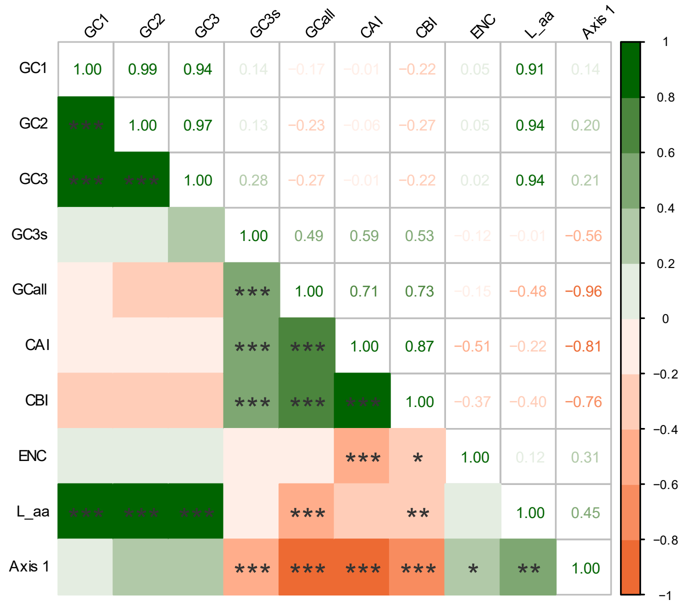

Ulva compressa MW548841

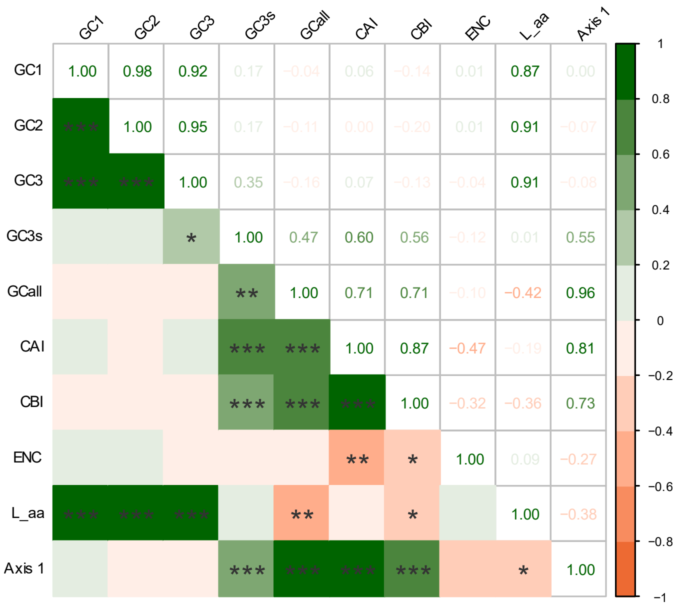

Ulva compressa MW353781

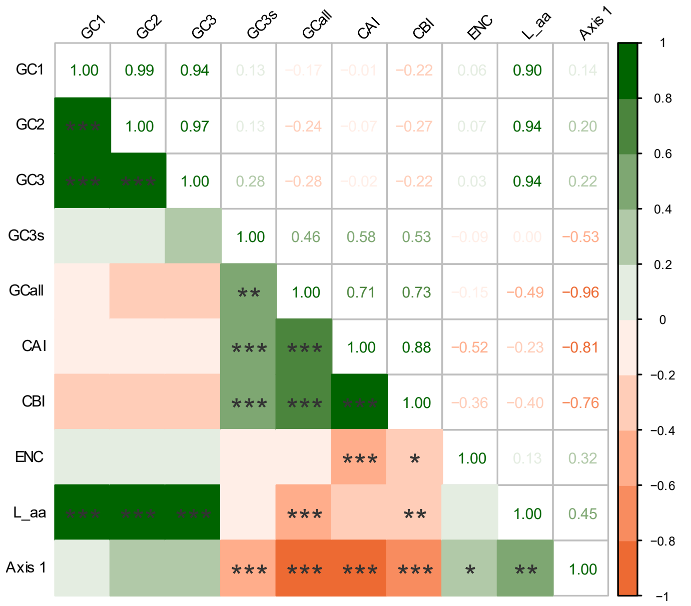

Ulva compressa MK069584

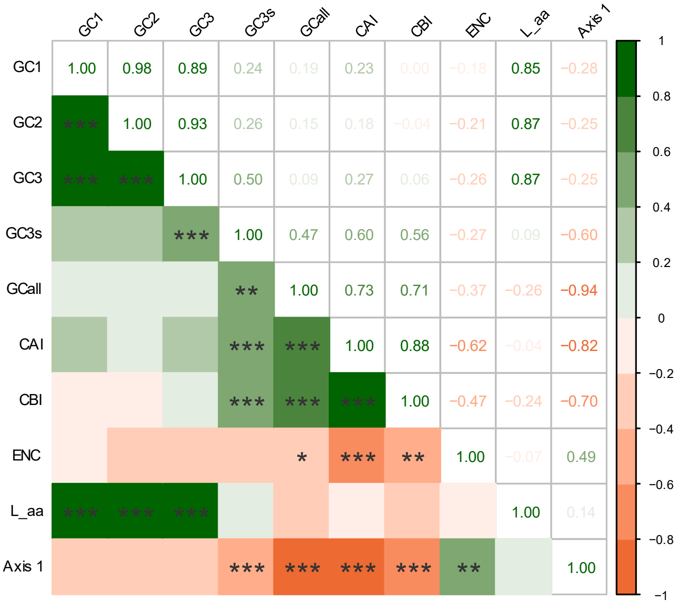

Ulva intestinalis MZ158703

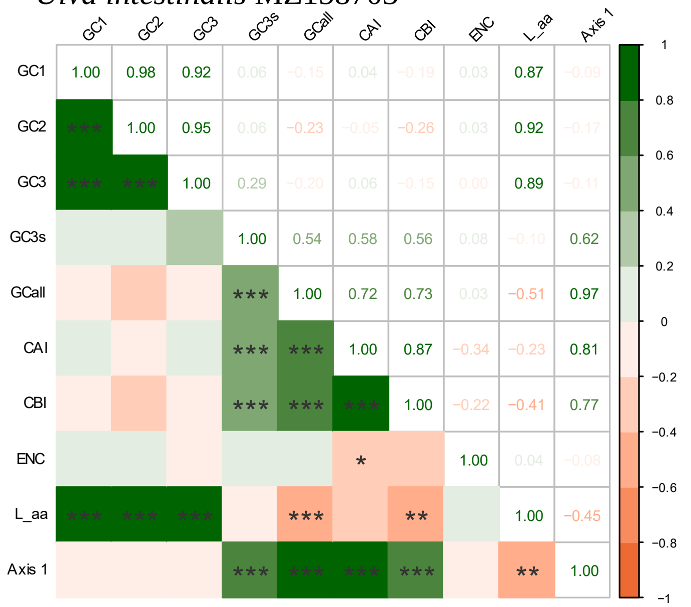

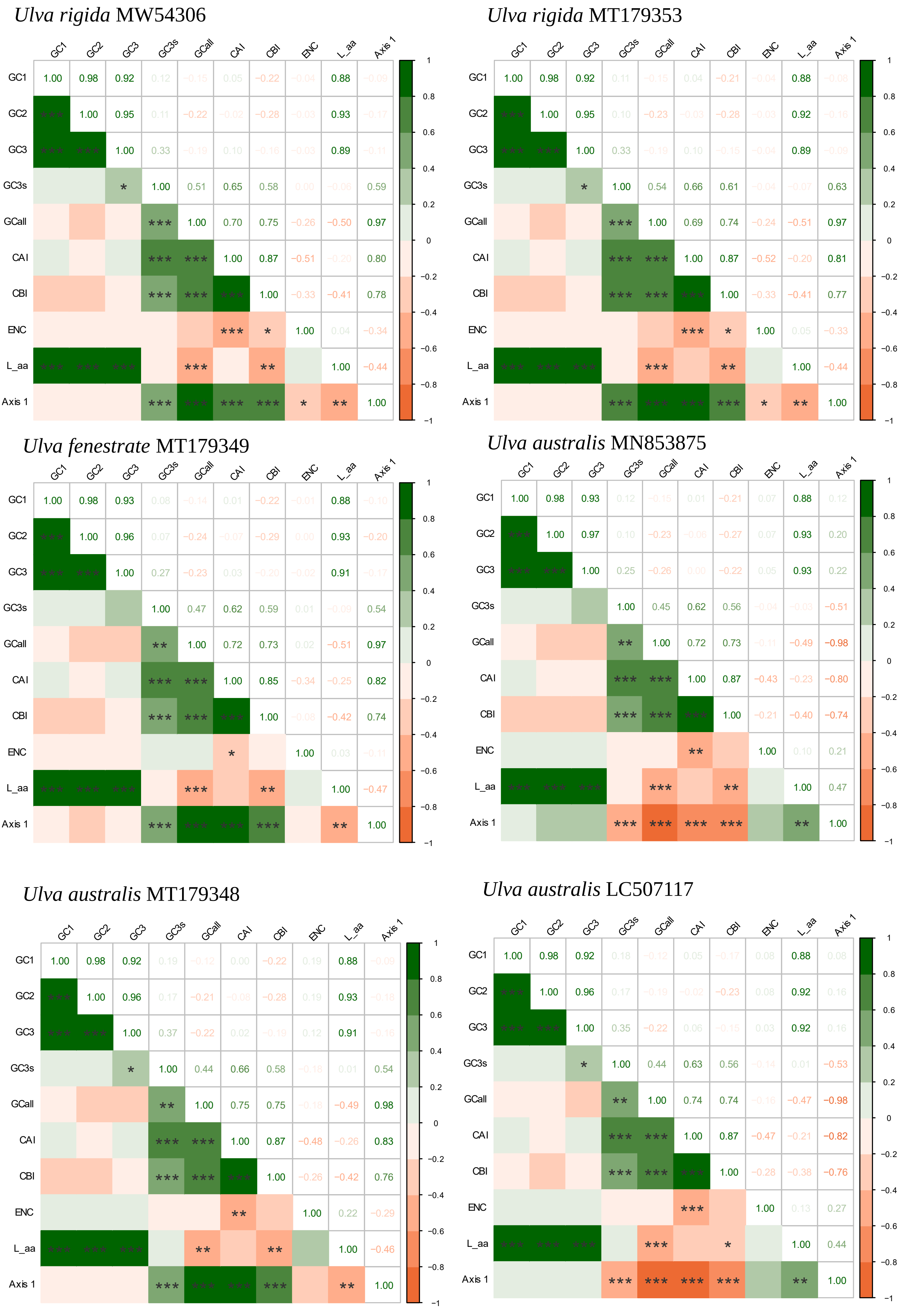

* P<0.05, ** P<0.01, *** P<0.001
